# Static and Dynamic Eosinophil Measures and In-Hospital Mortality in Patients with Sepsis: A Retrospective Cohort Study

**DOI:** 10.3390/medicina62040640

**Published:** 2026-03-27

**Authors:** Florentina Mușat, Octavian Andronic, Alexandra Bolocan, Cosmin-Alexandru Palcău, Alina Mihaela Dobre, Daniel Ion, Dan Nicolae Păduraru

**Affiliations:** 1 Faculty of Medicine, Carol Davila University of Medicine and Pharmacy, 050474 Bucharest, Romania; florentina.musat@drd.umfcd.ro (F.M.); bolocan.alexa@gmail.com (A.B.); alina.florian@gmail.com (A.M.D.); dr.daniel.ion@gmail.com (D.I.); dan.paduraru.nicolae@gmail.com (D.N.P.); 2 Innovation and eHealth Center, Carol Davila University of Medicine and Pharmacy Bucharest, 010451 Bucharest, Romania; 3 C.F.2 Clinical Hospital, 011464 Bucharest, Romania; alexandru-cosmin.palcau@drd.umfcd.ro; 4 General Surgery Department, University Emergency Hospital of Bucharest, 050098 Bucharest, Romania

**Keywords:** eosinophils, sepsis, mortality, dynamic, real-world study, retrospective, prediction

## Abstract

*Backgroundand Objectives*: Reliable prognostic biomarkers in sepsis that are inexpensive and universally available remain limited. Eosinophil counts decrease in systemic illness, but the prognostic value of static versus dynamic eosinophil measures in sepsis is uncertain. We investigated the association between eosinophil measurements, including early trajectories, and in-hospital mortality in a large sepsis cohort. *Materials and Methods*: We conducted a retrospective observational study of adult patients hospitalized with sepsis between 2021 and 2024 at a tertiary referral center. Absolute eosinophil counts were assessed at admission, at eosinophil nadir, and dynamically at approximately 48 and 72 h. Eosinophil trajectories were categorized as persistently undetectable, decrease, non-decrease, or rise from undetectable. Multivariable logistic regression models adjusted for demographic factors, comorbidities, and markers of disease severity were used to evaluate associations with in-hospital mortality. *Results*: Among 3932 patients, in-hospital mortality was 48.5%. Static eosinophil measures at admission and at eosinophil nadir were lower in non-survivors but were not independently associated with mortality after adjustment. Pancytopenia at eosinophil nadir was independently associated with increased mortality (OR 1.65, 95% CI 1.37–1.99). In contrast, eosinophil dynamics provided additional prognostic information. A decrease in eosinophil counts at 72 h was independently associated with higher mortality (OR 1.43, 95% CI 1.08–1.91), while 48 h changes were not. Persistently undetectable eosinophil trajectories were associated with the highest mortality, whereas recovery from undetectable levels was associated with lower risk. *Conclusions*: In sepsis, static eosinophil counts lack independent prognostic value, whereas delayed eosinophil decline and persistently suppressed trajectories are associated with increased in-hospital mortality. Eosinophil dynamics were associated with in-hospital mortality and may provide additional information on the host response during sepsis.

## 1. Introduction

Sepsis has been long recognized as a significant healthcare issue globally, associating high mortality, morbidity, and economic burden. While data from Central and Eastern Europe on the epidemiology and prognosis of sepsis remain limited, previous reports show that sepsis has a disproportionate impact incurring the heaviest burden on countries with limited resources [1,2]. In Romania, a large retrospective analysis, conducted in a regionally focused emergency hospital, revealed high rates of in-hospital mortality among patients with sepsis, suggesting a higher severity profile compared to that in Western Europe [3].

In such settings, early identification of patients at high risk of adverse outcomes is essential, but the use of advanced biomarkers or assays is often limited by cost, availability, and processing times. Consequently, there is a growing clinical interest in rapid and accessible prognostic stratification methods based on routine investigations available in any hospital. Parameters derived from the readily available standard complete blood count represent an attractive alternative for assessing the prognostic risk in patients with sepsis [4].

Eosinophils are a distinct leukocyte population, whose measurable numbers vary in response to systemic disease, reflecting alterations in immune response [5]. Although their depletion has not been associated with critical clinical consequences, reductions in eosinophil counts were found in states of severe illness and stress, making them potential disease-agnostic indicators of systemic inflammation [6].

Although eosinophils are not specific markers of infection, their circulating levels are highly sensitive to systemic stress responses and immune regulation during severe illness. For this reason, eosinopenia has repeatedly been proposed in the literature as a simple and widely available prognostic marker in patients with sepsis. However, the evidence remains inconsistent, and it is still unclear whether eosinophil measurements carry independent prognostic information once broader indicators of disease severity are taken into account [7,8]. Moreover, most previous studies have focused on single time-point measurements at admission, while the potential prognostic relevance of early eosinophil dynamics during hospitalization has been less systematically evaluated [7]. On the basis of these previous observations, we sought to understand whether variations in eosinophil counts could be used as a biomarker for anticipating disease severity and mortality in patients with sepsis.

Our approach was to investigate the prognostic value of both static and time-dependent eosinophil assessments in relation to in-hospital mortality in a large cohort of patients with sepsis. The analyses accounted for concurrent hematologic abnormalities and patient clinical characteristics, in order to determine whether eosinophil dynamics is independently related to mortality in sepsis.

## 2. Materials and Methods

### 2.1. Study Design and Data Source

This retrospective observational study was based on routinely collected electronic health records from the University Emergency Hospital of Bucharest (UEHB), a tertiary referral center and the largest emergency hospital in Romania. Clinical and laboratory data were extracted from the hospital information system, and all records were fully anonymized prior to analysis, in compliance with national and European data protection regulations. This study was designed and reported in accordance with the STROBE (Strengthening the Reporting of Observational Studies in Epidemiology) guidelines for observational studies. The completed STROBE checklist is provided in the Supplementary Materials.

### 2.2. Study Population and Sepsis Identification

**Figure 1:**
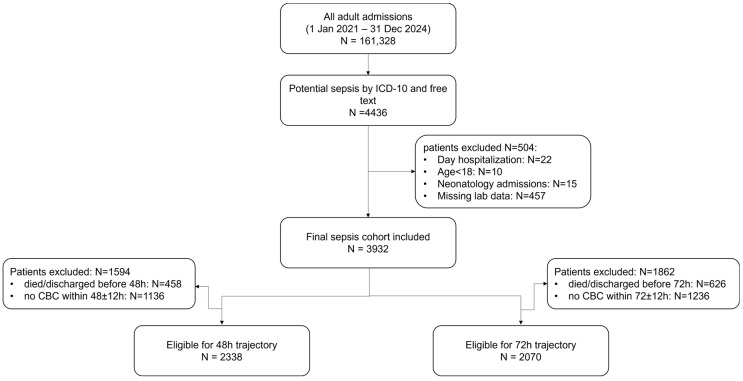
Patient selection and inclusion in the main cohort and in the 48 and 72 h eosinophil trajectory analyses.

All adult patients with sepsis admitted to the hospital between 1 January 2021, and 31 December 2024, were identified. Patients were identified using International Classification of Diseases (ICD)-10 codes compatible with sepsis, recorded as either primary or secondary diagnoses (Supplementary Table S1). In addition, a structured free-text search was performed within discharge diagnosis text fields using the terms “sepsis”, “septic”, and “septic shock”. The search was case-insensitive and applied using a substring matching rule (i.e., diagnoses containing these terms were identified). Consequently, composite terms such as “urosepsis” were also captured. No additional stemming algorithms, language variants, or misspelling corrections were applied. Although Sequential Organ Failure Assessment (SOFA) scores were not available for all admissions, patients without an explicit sepsis code were classified as septic only when documentation indicated infection (pyothorax, bronchopneumonia, peritonitis, gas gangrene, Clostridium difficile enterocolitis, endocarditis, bacterial meningoencephalitis, liver abscess, ischiorectal phlegmon/abscess, or necrotizing fasciitis) accompanied by acute organ dysfunction, in line with Sepsis-3 criteria (respiratory, cardiovascular, hepatic, coagulation, renal, or neurological). Given the retrospective administrative nature of the dataset, organ dysfunction was identified from coded diagnoses rather than recalculated physiologic SOFA values. The complete list of ICD-10 codes used for this administrative definition is provided in Supplementary Table S2. Patients younger than 18 years old, admissions to neonatology services, hospitalizations lasting <24 h, and patients with missing laboratory data required for analyses were excluded. After applying these criteria, 3932 patients were included in the final analysis (Figure 1).

Using this standardized approach, 3932 patients were included in the final cohort as follows: primary sepsis diagnosis (*n* = 1569), secondary sepsis diagnosis (*n* = 2115), free-text sepsis identification (*n* = 32), and sepsis inferred from infection plus organ dysfunction (*n* = 216). Because physiologic SOFA variables were not consistently available in the electronic dataset, sepsis identification relied on diagnostic codes and discharge diagnosis text fields. Therefore, this approach represents an administrative-data approximation of the Sepsis-3 definition rather than a direct physiologic implementation. Sepsis identification was based on diagnoses recorded during the index hospitalization, and the dataset did not allow reliable differentiation between sepsis present on admission and sepsis developing during hospitalization. However, the study was conducted in a large tertiary emergency hospital where most septic patients are admitted through the emergency department, making it likely that a substantial proportion of cases represent sepsis present at admission. For the purposes of the present analyses, T0 was defined as the first complete blood count obtained after hospital presentation.

Separate subsets were analyzed for 48 h and 72 h eosinophil trajectories; patients who died or were discharged before the respective time points or lacked complete blood count measurements within the predefined time windows were excluded. Of the 3932 patients in the main cohort, 2338 met eligibility criteria for the 48 h landmark analysis. Among excluded patients, 313 died before 48 h, 145 were discharged early, and 1136 lacked a complete blood count (CBC) within the predefined window. Similarly, 2070 patients were eligible for the 72 h analysis; exclusions were due to early death (*n* = 487), early discharge (*n* = 139), and missing CBC (*n* = 1236).

### 2.3. Hematological Time Points and Eosinophil Nadir

Complete blood count (CBC) data were retrieved for the entire duration of hospitalization. The admission value (T0) was defined as the first available CBC obtained after hospital presentation. Absolute eosinophil count (AEC), neutrophil, lymphocyte, and platelet counts were recorded as baseline values.

The eosinophil nadir was defined as the lowest AEC recorded during hospitalization. When multiple measurements shared the same minimum value, the earliest occurrence was retained. Absolute neutrophil, lymphocyte, and platelet counts were recorded at admission and at the time of eosinophil nadir.

### 2.4. Assessment of Early Eosinophil Change and Trajectory

Early eosinophil dynamics were evaluated by comparing AEC at admission (T0) with values measured at approximately 48 and 72 h after admission. Eosinophil values at 48 and 72 h were defined as the measurement closest to 48 and 72 h after admission, respectively, within a ±12 h window. Patients who died or were discharged before reaching the respective time points were excluded from the corresponding analyses. For each CBC obtained at time *t*, we calculated the absolute time distance (in hours) from the target landmark: distance_48_ = ∣(t−T0)×24−48∣. AEC (T72) was derived using the same procedure: distance_48_ = ∣(t−T0)×24−72∣.

The change in eosinophil count was calculated as ΔAEC = AEC(T0) − AEC(T48/T72), such that positive ΔAEC values indicated a decrease in eosinophil counts relative to baseline, whereas values ≤0 indicated stable or increased counts. This grouping was chosen to improve robustness in the presence of a pronounced measurement floor (frequent AEC = 0), where separating “stable” from “increase” yields small and potentially unstable categories.

For categorical analyses, patients were initially classified according to the direction of eosinophil change (decrease or no decrease). Four mutually exclusive eosinophil trajectory categories were defined separately for the 48 h and 72 h time points:

(1) persistently undetectable (AEC_T0 = 0 and AEC at follow-up = 0);
(2) decrease (AEC_T0 > 0 and AEC at follow-up < AEC_T0);
(3) non-decrease (AEC_T0 > 0 and AEC at follow-up ≥ AEC_T0); and
(4) rise from undetectable (AEC_T0 = 0 and AEC at follow-up > 0).

### 2.5. Hematological Indices and Pancytopenia

Eosinophil-derived ratios were calculated at both time points: eosinophil-to-neutrophil ratio (ENR), eosinophil-to-lymphocyte ratio (ELR), and eosinophil-to-platelet ratio (EPR) and each was evaluated in separate regression models.

Pancytopenia at eosinophil nadir was defined as the presence of at least two concurrent cytopenias among neutrophils, lymphocytes, and platelets measured at lowest recorded eosinophil value. Cytopenias were defined using conventional laboratory thresholds: neutrophil count < 1.5 × 10^9^/L, lymphocyte count < 1.0 × 10^9^/L, and platelet count < 150 × 10^9^/L. Pancytopenia was treated as a binary variable.

### 2.6. Study Outcome

The primary outcome was in-hospital mortality, defined as death occurring during the index hospitalization. Discharge status was extracted from the electronic medical records and coded as a binary outcome (survivor or non-survivor).

### 2.7. Statistical Analysis

Continuous variables were presented as mean ± SD and/or median with interquartile ranges (IQR), as appropriate and categorial variables as counts and percentages. Group comparisons were performed using the Mann–Whitney U test for continuous variables and χ^2^ or Fisher’s exact test for categorical variables, as appropriate. Comparisons across eosinophil trajectory groups were conducted using Kruskal–Wallis or χ^2^ tests.

Age- and sex-adjusted logistic regression models were used to screen candidate predictors. Multivariable logistic regression models were then constructed to assess the independent association between eosinophil-related variables and in-hospital mortality. Multivariable models included prespecified clinically relevant variables, such as mechanical ventilation, renal replacement therapy, intensive care unit (ICU) exposure (ICU admission or ICU length of stay, but not both simultaneously), and major comorbidities (cancer, pneumonia, cirrhosis, chronic kidney disease, and diabetes), regardless of screening significance. ICU admission, mechanical ventilation and renal replacement therapy were extracted as hospitalization-level indicators of severity; exact initiation timestamps were not available to restrict these variables strictly to the pre-landmark period. Key early physiologic severity markers commonly used in sepsis risk stratification (including serum lactate, vasopressor requirement, mean arterial pressure/hypotension, altered mental status, and early renal trajectory) were not consistently available in the electronic dataset and therefore could not be incorporated into the adjustment models. Pneumonia was defined based on ICD-10 codes for pneumonia or bronchopneumonia recorded during the index hospitalization; differentiation between community-acquired and hospital-acquired pneumonia was not consistently available in the dataset.

Collinearity among covariates was assessed prior to model fitting; highly correlated ICU-related variables were not entered simultaneously in the same model. ICU exposure can be operationalized either as a binary indicator (ICU admission) or as a continuous measure (ICU length of stay). Because ICU length of stay inherently subsumes ICU admission (values of 0 indicate no ICU stay, whereas values > 0 indicate ICU admission), these two variables are highly collinear and were therefore not entered simultaneously in multivariable models. ICU admission was used for descriptive/univariable comparisons, while ICU length of stay (hours) was used in multivariable models as a more granular proxy of illness severity and critical care burden.

As a sensitivity analysis, landmark models at 48 and 72 h were performed, including only patients alive and hospitalized at the respective time points. Because eosinophil trajectories at 48 and 72 h can only be defined in patients who survive and remain hospitalized until those time points, all trajectory models were fitted within landmark cohorts. Specifically, the 48 h models were restricted to patients alive and still hospitalized at 48 h with a CBC available within ±12 h (elig_48h = 1), and the 72 h models were restricted analogously (elig_72H = 1). All covariates used for adjustment were baseline or hospitalization-level variables available from the electronic record.

Several steps were taken to mitigate potential sources of bias. Confounding by illness severity was addressed through multivariable adjustment including prespecified clinical severity indicators and major comorbidities. To reduce survivorship and immortal-time bias in the dynamic analyses, eosinophil trajectories were evaluated using predefined 48 h and 72 h landmark cohorts restricted to patients alive and hospitalized at the respective time points. In addition, laboratory measurements were obtained from the same institutional platform using standardized procedures.

Because exact initiation timestamps for mechanical ventilation and renal replacement therapy were not available, the primary landmark models were specified without these variables to avoid potential post-landmark information leakage.

Separate models were fitted for 48 h and 72 h eosinophil dynamics. Results are reported as odds ratios (ORs) with 95% confidence intervals (95% CI). All statistical tests were two-sided, with *p* < 0.05 considered statistically significant. Continuous variables were analyzed on their original scale, and odds ratios are reported per unit increase. Visual inspection of associations with the log-odds of mortality suggested approximately monotonic relationships; therefore, no transformations were applied. Analyses were performed using Jamovi (version 2.7.13).

### 2.8. Ethical Approval

The study protocol conformed to the Declaration of Helsinki [9] and was approved by the Ethics Committee of the Carol Davila University of Medicine and Pharmacy (Approval No. 6098/21.03.2025) and by the Ethics Committee of the University Emergency Hospital of Bucharest (Approval No. 36964/17.06.2022).

## 3. Results

A total of 3932 adult patients hospitalized with sepsis were included in the final analysis (Table 1). The cohort was characterized by advanced age, high disease severity, and a substantial burden of comorbidities. More than one third of patients required ICU admission, and 30% required mechanical ventilation and/or renal replacement therapy.

Sepsis was recorded as the primary diagnosis in 1569 patients (40%) and as a secondary diagnosis in 2115 (54%), while an additional 248 patients (6%) were identified based on free-text documentation or evidence of infection associated with organ dysfunction. In-hospital mortality was high (48.5%), underscoring the severity of illness in the study population.

### 3.1. Static Eosinophil Analysis at Admission and at the Time of Eosinophil Nadir

When comparing survivors and non-survivors, marked differences were observed in both clinical and hematological profiles (Table 2). Non-survivors were older and exhibited greater disease severity, including more prolonged ICU exposure.

From a hematological perspective, non-survivors displayed a consistent pattern, characterized by higher neutrophil counts, and lower lymphocyte, thrombocytes and eosinophil counts compared to survivors. Eosinophil-derived ratios and the neutrophil-to-lymphocyte ratio at admission were also significantly altered in non-survivors.

Univariable associations between categorical clinical characteristics and in-hospital mortality are summarized in Table 3. Markers of disease severity, including mechanical ventilation, renal replacement therapy, and ICU admission, were strongly associated with increased mortality, as well as presence of comorbidities involving the liver, kidneys, and infections (cirrhosis, chronic kidney disease, pneumonia, and Clostridium difficile infection). Pancytopenia at the time of eosinophil nadir was more frequent among non-survivors and was also associated with increased in-hospital mortality. In contrast, surgical intervention and peritonitis (which is an indication for surgical intervention) were associated with lower odds of in-hospital death. Among the demographic characteristics, male sex was associated with lower odds of in-hospital death.

In models adjusted for severity markers and comorbidities, eosinophil nadir was not independently associated with mortality (Table 4). ICU stay, entered as a continuous variable, was not independently associated with mortality after adjustment.

In multivariate analysis, adjusted for age, sex, key severity indicators, and comorbidities, eosinophil derived ratios at admission were not significantly associated with in-hospital mortality in the multivariable model (Table 5).

Eosinophil-derived ratios calculated at the time of eosinophil nadir were not included in the main analyses because the near-universal occurrence of profound eosinopenia at nadir resulted in extreme or unstable estimates, limiting their interpretability and clinical relevance.

At the time of eosinophil nadir, 716 patients (18.2% of the cohort) met the criteria for pancytopenia, defined as the presence of at least two concurrent cytopenias. Among these patients, 419 (58.5%) died during hospitalization, compared with 297 (41.5%) survivors. In multivariable analysis, pancytopenia at eosinophil nadir remained independently associated with increased in-hospital mortality, whereas the absolute eosinophil count at nadir did not (Table 6).

### 3.2. Early Eosinophil Dynamics and In-Hospital Mortality

Given the limited independent prognostic value of static eosinophil measurements, we next examined whether early temporal changes in eosinophil counts convey additional prognostic information. Thus, we evaluated early dynamic changes in absolute eosinophil counts during the first 48 and 72 h after hospital admission. Because eosinophil trajectories require measurement at predefined time points, analyses at 48 and 72 h were necessarily restricted to patients who were alive, still hospitalized, and had an available CBC within the respective time windows. These analyses should therefore be interpreted as conditional (landmark-type) evaluations rather than time-zero predictions. Baseline characteristics of patients included versus excluded from the 48 h and 72 h analyses are shown in Supplementary Table S3. Among excluded patients, the majority lacked a repeat CBC within the 48 h window (71.3%), while a smaller proportion were excluded due to early death or discharge.

Absolute eosinophil counts at 48 and 72 h after admission were significantly lower in non-survivors compared with survivors (Table 7). At 48 h, survivors had a median eosinophil count of 0.1 × 10^9^/L (IQR 0–0.2), whereas non-survivors had a median of 0 × 10^9^/L (IQR 0–0.1; *p* < 0.001). Similar differences were observed at 72 h, with survivors showing higher eosinophil counts than non-survivors (*p* < 0.001). Changes in eosinophil counts from baseline (ΔAEC) also differed significantly between groups. Although median ΔAEC values were 0 at both 48 and 72 h in survivors and non-survivors, the distribution of changes differed significantly, with survivors exhibiting a wider interquartile range compared with non-survivors (*p* < 0.001 for both time points) (Table 7).

Although these differences reached statistical significance (*p* < 0.001), the distributions were characterized by zero medians and largely overlapping interquartile ranges, suggesting a limited clinical effect size and a potential floor effect related to the high prevalence of eosinopenia at baseline.

The direction of eosinophil changes within the first 48 and 72 h after admission was associated with in-hospital mortality (Table 8). At 72 h, patients who exhibited a decrease in eosinophil counts had a higher proportion of non-survivors compared with those without a decrease (18.9% versus 14.8%). A decrease in eosinophil counts at 72 h was significantly associated with increased in-hospital mortality (OR 1.34, 95% CI 1.07–1.69; *p* = 0.012). In contrast, at 48 h, although non-survivors more frequently exhibited a decrease in eosinophil counts than survivors (17.5% vs. 15.3%), this difference did not reach statistical significance (OR 1.17, 95% CI 0.94–1.46; *p* = 0.154).

To assess the robustness of the association, we performed separate multivariable logistic regression models using either the direction of eosinophil change (binary) or the absolute change in eosinophil count (continuous). In multivariable logistic regression analyses adjusting for age, sex, eosinophil count at admission, renal replacement therapy, mechanical ventilation, and relevant comorbidities, the direction of eosinophil change at 72 h remained independently associated with in-hospital mortality (Table 9). Patients who exhibited a decrease in eosinophil counts at 72 h had a significantly higher risk of in-hospital death compared with those without a decrease (OR 1.43, 95% CI 1.08–1.91; *p* = 0.014). In contrast, the direction of eosinophil change at 48 h was not independently associated with in-hospital mortality after multivariable adjustment (OR 1.18, 95% CI 0.90–1.56; *p* = 0.218). Stratified analyses by baseline detectability showed directionally consistent associations between eosinophil trajectories and in-hospital mortality at both 48 and 72 h (Supplementary Table S4).

However, the category of non-decrease includes a large proportion of patients who had a baseline eosinophil count of zero (median 0, IQR 0–0), which remained zero at 48 and 72 h. In contrast, patients with a decrease at 72 h had higher baseline eosinophil counts. For this reason, we further split the patients into four eosinophil trajectory groups: persistently undetectable, decrease, non-decrease, rise from undetectable.

**Figure 2:**
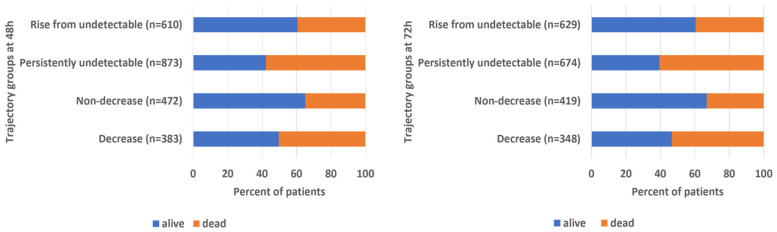
In-hospital outcome by eosinophil trajectory at 48 and 72 h. Bars show the proportion of survivors and non-survivors within each eosinophil trajectory group at each time point.

In-hospital mortality differed significantly across eosinophil trajectory groups at both 48 and 72 h after admission (Table 10; Figure 2). At both time points, persistently undetectable eosinophil counts were associated with the highest mortality, whereas non-decreasing trajectories were associated with the lowest mortality. Mortality among patients with a decrease in eosinophil counts was intermediate, while patients exhibiting a rise from undetectable levels showed outcomes closer to those of the non-decrease group. Overall differences across trajectory groups were statistically significant at both 48 and 72 h (*p* < 0.001). Notably, patients with a rise from undetectable eosinophil counts consistently exhibited lower mortality than those with persistently undetectable values, suggesting that early eosinophil recovery may reflect partial resolution of the underlying inflammatory response.

Trajectory models were estimated in landmark cohorts comprising 2338 patients at 48 h and 2070 patients at 72 h. In logistic regression analyses adjusted for clinical variables, eosinophil trajectory remained independently associated with in-hospital mortality at both 48 and 72 h after admission (Table 11). Using the non-decrease trajectory as the reference category, patients with a decrease in eosinophil counts had a significantly higher risk of in-hospital death at 48 h (OR 1.53, 95% CI 1.11–2.11; *p* = 0.0098), with an even stronger association observed at 72 h (OR 1.92, 95% CI 1.36–2.71; *p* < 0.001).

Persistently undetectable eosinophil counts were also independently associated with increased in-hospital mortality at both time points, with odds ratios of 1.85 (95% CI 1.41–2.42; *p* < 0.001) at 48 h and 2.41 (95% CI 1.79–3.25; *p* < 0.001) at 72 h. In contrast, a rise in eosinophil counts from undetectable levels was not independently associated with mortality at either 48 or 72 h. Among clinical covariates, increasing age, pneumonia, cirrhosis, renal replacement therapy, and mechanical ventilation were consistently associated with in-hospital mortality in both models. Sex, cancer, diabetes, and chronic kidney disease were not independently associated with mortality after adjustment.

Because exact initiation timestamps for mechanical ventilation and renal replacement therapy were not available, these variables could not be reliably restricted to the pre-landmark period. To minimize potential post-landmark information leakage, we therefore conducted additional models excluding these variables from the adjustment set; The results of these analyses are presented in Table 12.

In these models, the associations between eosinophil trajectories and in-hospital mortality remained directionally consistent with those observed in the primary analyses. Both the decrease trajectory and the persistently undetectable trajectory continued to show significant associations with increased mortality at 48 and 72 h, with effect estimates of similar or slightly greater magnitude compared with the main models. These findings indicate that the observed relationships between eosinophil trajectories and mortality are robust and not driven by adjustment for potential post-landmark covariates.

## 4. Discussion

Our findings showed that although static eosinophils counts at admission or at the time of eosinophil nadir were significantly lower among patients who died compared to patients who were alive during hospitalization, these were not independently associated with in-hospital mortality. These results further support the view that eosinophils are indicators of systemic immune state, rather than independent prognostic factors of adverse outcomes [5,10].

Emerging evidence indicates that eosinophils are active participants in host defense and immune regulation during infection, providing a biologically plausible link between eosinophil dynamics and sepsis outcomes [11]. Recent work highlights that eosinophils express diverse pattern-recognition and immunoglobulin receptors—including Toll-like receptors (TLR) TLR7, TLR2, Fc receptors, Siglec family members, cluster of differentiation (CD)—CD48 and CD300a—enabling pathogen sensing while incorporating inhibitory checkpoints that modulate activation [12,13,14,15].

In viral infections, recent models demonstrate direct antiviral activity. For example, eosinophils reduce viral burden in respiratory syncytial virus and parainfluenza infections via TLR7-driven nitric oxide production and granule protein release [16,17], while influenza studies show viral binding and enhancement of antiviral CD8^+^ T-cell responses [18]. However, context dependence is increasingly recognized: eosinophil recruitment can worsen airway pathology in some settings, and eosinopenia has been repeatedly associated with poorer outcomes in Coronavirus Disease-19 (COVID-19) [19], reinforcing the prognostic relevance of circulating eosinophil levels.

In bacterial infections—particularly relevant to sepsis—expanding data demonstrate both direct and indirect antimicrobial roles. Eosinophils can kill opsonized Escherichia coli [11] via extracellular deoxyribonucleic acid (DNA) traps and contribute to improved survival in polymicrobial sepsis models. Against Gram-positive pathogens, eosinophils generate reactive oxygen species (ROS) and degranulate in response to Staphylococcus aureus and its exotoxins via CD48 signaling, although live bacteria may induce eosinophil death [20]. Additional studies show eosinophil recruitment improves mucosal integrity and survival in Clostridioides difficile colitis through interleukin (IL)-25–dependent pathways [21], whereas in chronic Helicobacter pylori infection eosinophils may dampen Th1 responses and permit bacterial persistence [22].

Eosinopenia during acute infection is multifactorial and dynamic. Acute stress responses—mediated by endogenous corticosteroids, catecholamines, and inflammatory cytokines—promote rapid eosinophil apoptosis, margination to tissues, and bone marrow retention. Severe bacterial sepsis is also associated with redistribution of eosinophils to inflamed organs, increased consumption through degranulation, and suppression of eosinophilopoiesis by systemic inflammation. Iatrogenic factors, particularly systemic glucocorticoids and anti–IL-5/IL-5R biologics, further reduce circulating counts [11,23].

Importantly, the strong and independent association between pancytopenia at the time of eosinophil nadir and mortality further highlights that eosinopenia should be interpreted within the broader context of multilineage hematologic dysfunction. In accordance with our findings, other studies have shown how persistent suppression across multiple cell lines is robustly associated with adverse outcomes [24].

In contrast, the dynamic behavior of eosinophils over time was independently associated with mortality after adjustment, suggesting potential prognostic relevance that warrants formal incremental performance testing. The independent association between a decrease in eosinophil counts at 72 h and increased mortality is consistent with the hypothesis that persistent or recurrent eosinophil suppression better reflects ongoing immune dysregulation rather than an early, transient stress response. However, a similar association was not observed with 48 h measurements, underscoring the importance of timing in interpreting eosinophil counts in sepsis. Our results suggest that early eosinopenia is not a specific marker, whereas delayed recovery or further decline reflects the continuous state of systemic inflammation. The observation that patients with a rise from profound eosinopenia exhibited lower mortality shows how eosinophil re-emergence may signal partial restoration of immune homeostasis. The absence of an independent association at 48 h, contrasted with the stronger signal at 72 h, supports a time-dependent interpretation of eosinophil dynamics and motivated retention of both landmark analyses for comparative purposes.

Previous studies evaluating the role of eosinophils in predicting adverse outcomes in sepsis have predominantly focused on static measurements, most commonly absolute eosinophil counts at hospital admission. While a consistent unadjusted association between lower admission eosinophil counts and mortality has been reported, particularly in COVID-19 cohorts, substantial heterogeneity exists with respect to eosinophil reporting, analytical approaches, and the definition of eosinopenia, with cut-off values ranging from undetectable levels to 50 cells/mm^3^. This methodological variability has contributed to high statistical heterogeneity in pooled analyses and partly explains the conflicting conclusions reported across studies [7,10,25,26]. A meta-analysis performed by Lin and colleagues comprising 3842 subjects with sepsis from 7 studies, found that eosinopenia was highly prevalent among patients with sepsis and could potentially be a useful diagnostic marker. However, the authors noted limitations due to high heterogeneity across studies and the absence of a consistent eosinophil cutoff. Moreover, most of the available evidence was based on admission eosinophil counts and did not address prognostic implications or longitudinal eosinophil behavior [10].

In contrast, data examining longitudinal eosinophil changes suggest that temporal patterns may carry greater prognostic relevance than single time-point measurements, with meaningful separation between survivors and non-survivors often emerging between days 2 and 5 of hospitalization. Shravani et al. reported in a study on 100 septic patients that declining eosinophil counts during the first 72 h of hospitalization were associated with increased mortality and organ failure, whereas admission eosinophil values showed limited prognostic utility. Their findings supported a time-dependent interpretation of eosinophil behavior but were derived from a small, single-center cohort and restricted to patients surviving beyond 72 h, and may not be generalizable [27].

However, two studies have reported limited predictive value of eosinophils when incorporated into multivariable models dominated by age, comorbidities, and markers of organ failure, reinforcing the interpretation of eosinophils as indicators of global immune dysregulation rather than independent prognostic drivers [27,28].

Anoop et al. reported that admission eosinophil counts were strongly associated with in-hospital mortality in patients with sepsis and underlying cirrhosis, with a cutoff of AEC < 110/mm^3^; however, this association was observed in a highly selected population with advanced cirrhosis and relied exclusively on static admission measurements, without consideration of eosinophil dynamics or broader hematologic context [28].

Our study has several important strengths, including the large sample size, the inclusion of an unselected and clinically heterogeneous sepsis population, and the comprehensive evaluation of eosinophil behavior using both static measurements and early temporal trajectories. The availability of detailed hematologic data allowed for the assessment of eosinophil dynamics in the context of concurrent multilineage cytopenias and major clinical indicators of disease severity, providing a more nuanced interpretation of eosinophil-related prognostic signals.

However, several limitations should be acknowledged. The retrospective observational design precludes causal inference and is subject to residual confounding despite extensive multivariate adjustment. Analyses of eosinophil dynamics were necessarily restricted to patients surviving beyond 48 and 72 h, introducing potential survivorship bias.

Because eosinophil trajectory classification required survival and continued hospitalization to the respective time points, the dynamic analyses are inherently subject to survivorship and immortal-time bias. As shown in Supplementary Table S3, patients excluded before 48 and 72 h experienced substantially higher early mortality. Therefore, trajectory findings should be interpreted as conditional associations rather than time-zero prognostic effects. Additionally, the absence of follow-up CBC measurements in a substantial proportion of patients likely reflects real-world variation in monitoring intensity and may introduce selection bias.

Because the exact timing of mechanical ventilation and renal replacement therapy initiation was unavailable, these variables may partially reflect downstream disease severity rather than purely baseline confounding, and some degree of overadjustment cannot be excluded.

The absence of granular physiologic severity indicators may result in residual confounding by unmeasured illness severity despite adjustment for available proxies such as ICU exposure, mechanical ventilation, and renal replacement therapy.

We did not formally assess incremental prognostic performance (e.g., change in discrimination or reclassification) beyond multivariable adjustment, and therefore our findings should be interpreted as associative rather than as evidence of clinical prediction utility.

Additionally, the high prevalence of profound eosinopenia resulted in a floor effect that may limit the clinical interpretability of absolute eosinophil changes. Finally, eosinophil measurements were not obtained at standardized time points, and mechanistic data were unavailable, limiting insight into the underlying biological pathways linking eosinophil dynamics to adverse outcomes. Future studies incorporating standardized longitudinal sampling and immune phenotyping may help clarify whether eosinophil recovery reflects resolution of systemic inflammation or broader immune reconstitution.

## 5. Conclusions

In this large cohort of hospitalized patients with sepsis, static eosinophil measurements at admission or at eosinophil nadir were not independently associated with in-hospital mortality after adjustment for disease severity and concurrent hematologic abnormalities. In contrast, persistent or delayed eosinophil decline at 72 h was independently associated with increased mortality, highlighting the time-dependent nature of eosinophil behavior in sepsis. Importantly, the prognostic relevance of eosinophil dynamics emerged in conjunction with markers of global hematologic dysfunction, particularly pancytopenia, supporting the interpretation of eosinophils as indicators of systemic immune dysregulation rather than isolated prognostic biomarkers. These findings help reconcile previously conflicting results in the literature, since eosinophil trajectories were associated with in-hospital mortality, but further studies are needed to determine whether these associations translate into clinically useful prediction models or risk stratification tools.

## Figures and Tables

**Table 1:** Baseline characteristics of the study population.

Dataset Characteristics	*n* = 3932
Mean age (SD)	70.09 (14.84)
	Women: 72.89 (14.91)
	Men: 67.23 (14.21)
Median age (range, IQR)	72 (18–102)
	Women: 75 (18–102)
	Men: 69 (18–100)
Women, *n* (%)	1982 (50%)
Men, *n* (%)	1950 (50%)
Sepsis as main diagnosis, *n* (%)	1569 (40%)
Sepsis as secondary diagnosis, *n* (%)	2115 (54%)
Sepsis as free-text diagnosis or infection associated with organ dysfunction, *n* (%)	248 (6%)
Only mechanical ventilation, *n* (%)	736 (19%)
Only renal Replacement therapy, *n* (%)	75 (2%)
Mechanical ventilation and renal replacement, *n* (%)	358 (9%)
No mechanical ventilation or renal replacement, *n* (%)	2763 (70%)
Surgical cases, *n* (%)	968 (25%)
Non-surgical cases, *n* (%)	2964 (75%)
Any cancer, *n* (%)	692 (18%)
Hematological cancer, *n* (%)	150 (4%)
Non-hematological cancer, n (%)	542 (14%)
Diabetes, *n* (%)	1156 (29%)
Cirrhosis, *n* (%)	189 (5%)
Chronic kidney disease, *n* (%)	758 (19%)
Chronic obstructive pulmonary disease, *n* (%)	258 (7%)
Clostridium difficile infection, *n* (%)	316 (8%)
Atherosclerosis, *n* (%)	496 (13%)
Peritonitis, *n* (%)	266 (7%)
Pneumonia, *n* (%)	1017 (26%)
In-hospital mortality, *n* (%)	1908 (49%)
Mean hospitalization, days (SD, *n* = 3932)	13.83 (14.8)
Median hospitalization, days (range, *n* = 3932)	10 (0–213)
Patients with ICU admission, *n* (%)	1444 (37%)
ICU stay, hours, mean (SD, *n* = 1444)	194.18 (269.22)
ICU stay, hours, median (range, *n* = 1444)	94.01 (2.48–3110.42)

Abbreviations: ICU, intensive care unit; SD, standard deviation; IQR, interquartile range.

**Table 2:** Comparison of clinical and hematological parameters between survivors and non-survivors.

Variable	Survivors (Median [IQR])	Non-Survivors (Median [IQR])	*p* Value (Mann–Whitney)
Age	69 (58–78)	75 (66–84)	**<0.001**
Hospitalization, days	11 (7–18)	7 (2–16)	**<0.001**
ICU stay, hours	0 (0–12.662)	0 (0–99.912)	**<0.001**
Minimum Neutrophil count	5.1 (3.4–7.225)	7.2 (4–12.2)	**<0.001**
Neutrophil count at admission	11 (6.7–16.1)	11.600 (7.1–17.4)	0.00148
Minimum Lymphocyte count	0.7 (0.4–1.2)	0.500 (0.3–0.8)	**<0.001**
Lymphocyte count at admission	1 (0.6–1.5)	0.800 (0.5–1.4)	**<0.001**
Minimum Eosinophil count	0 (0–0.1)	0 (0–0)	**<0.001**
Maximum Eosinophil count	0.3 (0.1–0.5)	0.100 (0–0.4)	**<0.001**
Eosinophil count at admission	0 (0–0.1)	0 (0–0.1)	**<0.001**
Mean Eosinophil count	0.122 (0.058–0.216)	0.05 (0–0.125)	**<0.001**
Minimum Platelet count	179 (111–260)	117.000 (50–197)	**<0.001**
Platelet count at admission	231 (157–330)	209.000 (135–299)	**<0.001**
ENR at admission	0 (0–0.013)	0 (0–0.00705)	**<0.001**
ELR at admission	0 (0–0.105)	0 (0–0.076)	**<0.001**
NLR at admission	10.5 (5.545–19.333)	12.891 (7–24)	**<0.001**
EPR at admission	0 (0–0.00052)	0 (0–0.0004)	**<0.001**

Abbreviations: ICU, intensive care unit; IQR, interquartile range; ENR, eosinophil-to-neutrophil ratio; ELR, eosinophil-to-lymphocyte ratio; EPR, eosinophil-to-platelet ratio; NLR, neutrophil-to-lymphocyte ratio. Cell counts are expressed as ×10^9^/L.

**Table 3:** Univariable associations between clinical characteristics and in-hospital mortality.

Clinical Characteristics	Survivors *n* (%)	Non-Survivors *n* (%)	OR (95% CI)	*p* Value (χ^2^/Fisher)
Male sex	1051 (51.9%)	899 (47.1%)	0.825 (0.728–0.935)	0.0026
Mechanical ventilation	221 (10.9%)	873 (45.8%)	6.88 (5.83–8.13)	**<0.001**
Renal replacement therapy	97 (4.8%)	336 (17.6%)	4.25 (3.36–5.37)	**<0.001**
ICU admission	530 (25.2%)	914 (47.9%)	2.59 (2.27–2.96)	**<0.001**
Surgical intervention	607 (30.0%)	361 (18.9%)	0.545 (0.469–0.632)	**<0.001**
Any cancer	336 (16.6%)	349 (18.3%)	1.12 (0.954–1.33)	0.162
Hematological cancer	80 (4%)	70 (3.7%)	0.925 (0.667–1.28)	0.642
Non-hematological cancer	258 (12.7%)	284 (14.9%)	1.2 (0.998–1.44)	0.0520
Diabetes	616 (30.4%)	540 (28.3%)	0.902 (0.786–1.04)	0.142
Cirrhosis	75 (3.7%)	114 (6%)	1.65 (1.23–2.23)	**<0.001**
Chronic kidney disease	294 (14.5%)	464 (24.3%)	1.89 (1.61–2.22)	**<0.001**
Chronic obstructive pulmonary disease	118 (5.8%)	140 (7.3%)	1.28 (0.993–1.65)	0.0564
Clostridium difficile infection	140 (6.9%)	176 (9.2%)	1.37 (1.09–1.72)	0.00782
Atherosclerosis	275 (13.6%)	221 (11.6%)	0.883 (0.689–1.01)	0.0585
Peritonitis	168 (8.3%)	98 (5.1%)	0.598 (0.462–0.774)	**<0.001**
Pneumonia	339 (16.7%)	678 (35.5%)	2.74 (2.36–3.18)	**<0.001**
Pancytopenia	297 (14.7%)	419 (22.0%)	1.64 (1.39–1.93)	**<0.001**

Abbreviations: ICU, intensive care unit; OR, odds ratio; CI, confidence interval.

**Table 4:** Multivariable logistic regression model adjusted for clinical variables including eosinophil nadir and concurrent blood cell lines.

Predictor	*p* Value	OR (95% CI)
Sex	0.827	0.984 (0.848–1.141)
Age	**<0.001**	1.045 (1.039–1.051)
Mechanical ventilation	**<0.001**	7.324 (5.899–9.093)
Renal replacement therapy	**<0.001**	1.762 (1.311–2.366)
Any cancer	0.015	1.268 (1.046–1.537)
Diabetes	0.009	0.807 (0.687–0.949)
Pneumonia	**<0.001**	1.842 (1.546–2.194)
Chronic kidney disease	0.180	1.140 (0.941–1.382)
Platelet count at eosinophil nadir	**<0.001**	0.998 (0.897–0.998)
Minimum eosinophil count	0.363	0.820 (0.535–1.257)
Neutrophil count at eosinophil nadir	**<0.001**	1.028 (1.018–1.037)
Lymphocyte count at eosinophil nadir	0.247	0.991 (0.975–1.007)
ICU stay, hours	0.418	1.000 (0.999–1.000)

Abbreviations: CI, confidence interval; ICU, intensive care unit; OR, odds ratio.

**Table 5:** Multivariable models adjusted clinical variables for eosinophil-derived ratios at admission.

Variable	OR (95% CI)	*p* Value
ENR at admission	0.527 (0.083–3.361)	0.498
ELR at admission	0.932 (0.831–1.047)	0.235
EPR at admission	4.175 (0.041–427.502)	0.545

Abbreviations: ENR, eosinophils/neutrophils; ELR, eosinophils/lymphocytes; EPR, eosinophils/platelets; OR, odds ratio.

**Table 6:** Model adjusted for clinical variables assessing the association between pancytopenia at eosinophil nadir and mortality.

Predictor	*p* Value	OR (95% CI)
Sex	0.748	0.976 (0.842–1.131)
Age	**<0.001**	1.048 (1.042–1.054)
Mechanical ventilation	**<0.001**	7.201 (5.812–8.922)
Renal replacement therapy	**<0.001**	1.840 (1.372–2.461)
Any cancer	0.094	1.177 (0.973–1.423)
Diabetes	0.0005	0.794 (0.677–0.932)
Pneumonia	**<0.001**	1.820 (1.529–2.166)
Chronic kidney disease	0.205	1.131 (0.935–1.370)
ICU stay, hours	0.385	1.000 (0.999–1.000)
Minimum eosinophil count	0.905	1.029 (0.645–1.642)
Pancytopenia	**<0.001**	1.648 (1.368–1.986)

Abbreviations: ICU, intensive care unit; OR, odds ratio.

**Table 7:** Absolute eosinophil counts and early changes at 48 and 72 h in survivors and non-survivors.

Variable	Survivors, *n*	Survivors: Median (IQR)	Non-Survivors, *n*	Non-Survivors: Median (IQR)	*p* Value (Mann–Whitney)
AEC at 48 h (×10^9^/L)	1235	0.1 (0–0.2)	1103	0.0 (0–0.1)	**<0.001**
AEC at 72 h (×10^9^/L)	1088	0.1 (0–0.2)	982	0.0 (0–0.1)	**<0.001**
ΔAEC at 48 h (×10^9^/L)	1235	0 (−0.1–0)	1103	0 (−0.1–0)	**<0.001**
ΔAEC at 72 h (×10^9^/L)	1088	0 (−0.2–0)	982	0 (−0.1–0)	**<0.001**

Abbreviations: AEC, absolute eosinophil count (×10^9^/L); IQR, interquartile range.

**Table 8:** Direction of eosinophil change within 48 and 72 h and in-hospital mortality.

Variable	Group	Survivors, n (%)	Non-Survivors, *n* (%)	OR (95% CI)	*p* (χ^2^/Fisher)
Direction of eosinophil change at 72 h	No decrease (ΔAEC ≤ 0)	926 (85.2%)	796 (81.1%)	Reference	0.012
	With decrease (ΔAEC > 0)	161 (14.8%)	186 (18.9%)	1.34 (1.07–1.69)	
Direction of eosinophil change at 48 h	No decrease (ΔAEC ≤ 0)	1045 (84.7%)	910 (82.5%)	Reference	0.154
	With decrease (ΔAEC > 0)	189 (15.3%)	193 (17.5%)	1.17 (0.94–1.46)	

Abbreviations: AEC, absolute eosinophil count; CI, confidence interval; OR, odds ratio.

**Table 9:** Multivariable logistic regression evaluating the association between the direction of eosinophil change at 48 and 72 h and in-hospital mortality, adjusted for age, sex, absolute eosinophil count at admission, renal replacement therapy, mechanical ventilation and relevant comorbidities (any cancer, diabetes, pneumonia, cirrhosis, chronic kideny disease).

Predictor	OR (95% CI)	*p* Value
**Direction of change 72 h** **(decrease vs. no decrease)**	1.432 (1.075–1.906)	0.014
**Direction of change 48 h** **(decrease vs. no decrease)**	1.18 (0.90–1.56)	0.218

Abbreviations: CI, confidence interval; OR, odds ratio.

**Table 10:** In-hospital mortality according to eosinophil trajectory at 48 h and at 72 h after admission.

**Eosinophil trajectory at 48 h**	**In-hospital mortality** ***n*** **(%)**	***p*** **< 0.001**
Decrease (*n* = 383)	193 (50.4)	
Non-decrease (*n* = 472)	165 (35.0)	
Persistently undetectable (*n* = 873)	504 (57.7)	
Rise from undetectable (*n* = 610)	241 (39.5)	
**Eosinophil trajectory at 72 h**	**In-hospital mortality** ***n*** **(%)**	***p*** **< 0.001**
Decrease (*n* = 348)	186 (53.4)	
Non-decrease (*n* = 419)	139 (33.2)	
Persistently undetectable (*n* = 674)	408 (60.5)	
Rise from undetectable (*n* = 629)	249 (39.6)	

**Table 11:** Logistic regression analyses adjusted for clinical variables evaluating the association between eosinophil trajectory at 48 and 72 h and in-hospital mortality.

Predictor	OR (95% CI)—48 h	*p* Value	OR (95% CI)—72 h	*p* Value
Decrease vs. non-decrease	1.529 (1.107–2.110)	0.0098	1.921 (1.363–2.706)	**<0.001**
Persistently undetectable vs. non-decrease	1.846 (1.406–2.424)	**<0.001**	2.409 (1.787–3.248)	**<0.001**
Rise from undetectable vs. non-decrease	1.075 (0.804–1.437)	0.624	1.128 (0.833–1.526)	0.436
Age (per year)	1.047 (1.038–1.055)	**<0.001**	1.045 (1.036–1.053)	**<0.001**
Male sex	0.979 (0.804–1.190)	0.827	0.843 (0.681–1.042)	0.114
Cancer	1.205 (0.939–1.545)	0.143	1.189 (0.904–1.563)	0.215
Pneumonia	1.844 (1.479–2.297)	**<0.001**	1.948 (1.537–2.468)	**<0.001**
Cirrhosis	2.155 (1.390–3.341)	**<0.001**	2.087 (1.319–3.302)	0.001
Chronic kidney disease	1.158 (0.904–1.483)	0.244	1.265 (0.969–1.652)	**0.083**
Diabetes	0.957 (0.776–1.178)	0.677	0.889 (0.707–1.118)	0.315
Renal replacement therapy	1.938 (1.396–2.691)	**<0.001**	1.999 (1.415–2.823)	**<0.001**
Mechanical ventilation	6.832 (5.370–8.691)	**<0.001**	6.887 (5.351–8.864)	**<0.001**

Abbreviations: CI, confidence interval; OR, odds ratio; vs., versus.

**Table 12:** Sensitivity analyses of landmark trajectory models at 48 and 72 h excluding mechanical ventilation and renal replacement therapy from adjustment.

Predictor	OR (95% CI)—48 h	*p* Value	OR (95% CI)—72 h	*p* Value
Decrease vs. non-decrease	1.782 (1.331–2.385)	**<0.001**	2.206 (1.62–3.00)	**<0.001**
Persistently undetectable vs. non-decrease	2.1245 (1.66–2.72)	**<0.001**	2.655 (2.025–3.481)	**<0.001**
Rise from undetectable vs. non-decrease	1.162 (0.893–1.513)	0.262	1.26 (0.957–1.656)	0.099
Age (per year)	1.028 (1.021–1.035)	**<0.001**	1.024 (1.017–1.032)	**<0.001**
Male sex	0.94 (0.785–1.122)	0.488	0.797 (0.658–0.967)	0.021
Cancer	1.236 (0.985–1.55)	0.066	1.287 (1.005–1.649)	0.045
Pneumonia	2.897 (2.38–3.526)	**<0.001**	3.188 (2.585–3.932)	**<0.001**
Cirrhosis	2.02 (1.353–3.017)	**<0.001**	1.767 (1.163–2.686)	0.007
Chronic kidney disease	1.74 (1.398–2.166)	**<0.001**	1.895 (1.5–2.397)	**<0.001**
Diabetes	1.03 (0.852–1.244)	0.761	0.99 (0.806–1.217)	0.930

Abbreviations: CI, confidence interval; OR, odds ratio; vs., versus.

## Data Availability

The datasets generated and/or analyzed during the current study are not publicly available due to institutional data protection regulations but are available from the corresponding author upon reasonable request and with permission of the University Emergency Hospital of Bucharest.

## Supplementary Materials

The following supporting information can be downloaded at: https://www.mdpi.com/article/10.3390/medicina62040640/s1, Table S1: Distribution of sepsis cases according to ICD-10 diagnostic codes in the study cohort. Values represent the number of patients in whom the respective ICD-10 code was identified in the hospital discharge diagnoses, either as the primary or as a secondary diagnosis; Table S2: ICD-10 codes used to identify acute organ dysfunction in the administrative Sepsis-3–aligned identification pathway. SOFA—Sequential Organ Failure Assessment; ICD—International Classification of Diseases; ARDS –Acute respiratory distress syndrome; DIC—Disseminated intravascular coagulopathy; Table S3: Baseline characteristics of patients included versus excluded from the 48 h (A) and 72 h (B) eosinophil trajectory analysis; Table S4: Sensitivity analyses stratified by baseline eosinophil detectability. A. At 48 h. B. At 72 h; Table S5: STROBE Statement—Checklist of items that should be included in reports of cohort studies.
