# Supplementary material for: Static and Dynamic Eosinophil Measures and In-Hospital Mortality in Patients with Sepsis: A Retrospective Cohort Study
Source: Medicina (Kaunas). 2026 Mar 27;62(4):640. doi: 10.3390/medicina62040640 (PMC13118259; doi:10.3390/medicina62040640)
Supplement: Supplementary file 1 [file medicina-62-00640-s001.zip › medicina-4164961-supplementary.pdf]

Supplementary Table S1. Distribution of sepsis cases according to ICD-10 diagnostic codes in the study cohort. Values represent the number of patients in whom the respective ICD-10 code was identified in the hospital discharge diagnoses, either as the primary or as a secondary diagnosis.

		Total
A02.1	Sepsis due to Salmonella	3
A26.7	Sepsis due to Erysipelothrix	0
A32.7	Listerian sepsis	0
A40.0	Sepsis due to Group A Streptococcus	0
A40.1	Sepsis due to Group B Streptococcus	2
A40.2	Sepsis due to Group D Streptococcus	3
A40.8	Other types of sepsis due to Streptococci	13
A40.9	Unspecified streptococcal sepsis	4
A41.0	Sepsis due to Staphylococcus aureus	37
A41.1	Sepsis due to other specified staphylococci	34
A41.2	Sepsis due to other unspecified staphylococci	7
A41.3	Sepsis due to Haemophilus influentzae	2
A41.4	Sepsis due to anaerobes	15
A41.50	Sepsis due to unspecified Gram-negative bacteria	22
A41.51	Sepsis due to Escherichia coli	89
A41.52	Sepsis due to pseudomonas	26
A41.58	Sepsis due to other Gram-negative organisms	61
A41.8	Other specified types of sepsis	2373
A41.9	Unspecified sepsis	1105
A42.7	Actinomycotic sepsis	3
B37.7	Sepsis due to Candida	20
T81.42	Procedure-related sepsis	4
O88.3	Obstetric pyemic and septic embolism	0
	TOTAL	3823

Supplementary Table S2. ICD-10 codes used to identify acute organ dysfunction in the administrative Sepsis-3–aligned identification pathway. SOFA- Sequential Organ Failure Assessment; ICD - International Classification of Diseases; ARDS –Aacute respiratory distress syndrome; DIC – Disseminated intravascular coagulopathy.

**Organ system (SOFA domain)**	**ICD-10 codes**	**Description**
Respiratory	J96.0, J96.9, J80	Acute respiratory failure, ARDS

Cardiovascular	R57.0, R57.1, R57.8, R57.9	Shock
Renal	N17.0, N17.1, N17.2, N17.9	Acute kidney failure
Hepatic	K72.0	Acute hepatic failure
Neurologic	G93.4	Encephalopathy
Coagulation	D65, D69.6	DIC, thrombocytopenia

Supplementary Table S3. Baseline characteristics of patients included versus excluded from the 48-hour (A) and 72-hours (B) eosinophil trajectory analysis. Patients were classified as included if they were alive, still hospitalized, and had an available complete blood count within the predefined 48-hour window (±12 h). Continuous variables are presented as median (interquartile range) and were compared using the Mann–Whitney U test. Categorical variables are presented as counts (percentages) and were compared using the χ^2 test. Early death <48 h was defined as in-hospital death occurring within the first 48 hours from admission.

A.

	**Included at 48h**	**Excluded at 48h**	**p value**
**N**	2338	1594	
**Age, median (IQR)**	72 (62-81)	72 (63-82.8)	0.015
**baseline AEC ×10^9/L, median (IQR)**	0 (0-0.1)	0 (0-0.1)	<0.001
**ICU admission, n (%)**	1059 (45.29%)	385 (24.15%)	<0.001
**Mechanical ventilation, n (%)**	782 (33.44%)	312 (19.57%)	<0.001
**RRT, n (%)**	338 (14.45%)	95 (5.95%)	<0.001
**Death within 48 h, n (%)**	0 (0.0%)	313 (19.6%)	<0.001

B.

	**Included at 72h**	**Excluded at 72h**	**p value**
**N**	2070	1862	
**Age, median (IQR)**	72 (62-81)	72 (63-81)	0.161
**Baseline AEC ×10^9/L, median (IQR)**	0 (0-0.1)	0 (0-0.1)	0.013
**ICU admission, n (%)**	991 (47.87%)	453 (24.32%)	<0.001
**Mechanical ventilation, n (%)**	746 (36.03%)	348 (18.68%)	<0.001
**RRT, n (%)**	314 (15.16%)	119 (6.39%)	<0.001
**Death within 72h, n (%)**	0 (0.0%)	487 (26.2%)	<0.001

Abbreviations: ICU, intensive care unit; RRT, renal replacement therapy; AEC, absolute eosinophil count (×10^9/L); IQR, interquartile range.

Supplementary Table S4. Sensitivity analyses stratified by baseline eosinophil detectability. A. At 48h. B. At 72h.

A. At 48h

**Stratum**	**Comparison**	**N**	**Adjusted OR (95% CI)**	**p**
AEC_T0 = 0	Persistently undetectable vs Rise from undetectable	1483	0.583 (0.457–0.744)	<0.001
AEC_T0 > 0	Non-decrease vs Decrease	855	1.520 (1.094–2.112)	0.013

B. At 72h

**Stratum**	**Comparison**	**N**	**Adjusted OR (95% CI)**	**p**
AEC_T0 = 0	Persistently undetectable vs Rise from undetectable	1303	0.469 (0.361–0.609)	<0.001
AEC_T0 > 0	Decrease vs Non-decrease	767	0.525 (0.371–0.741)	<0.001

Supplementary Table S5 STROBE Statement—Checklist of items that should be included in reports of cohort studies

	**Item No**	**Recommendation**
**Title and abstract**	1<br>Page1	(*a*) Indicate the study's design with a commonly used term in the title or the abstract
		(*b*) Provide in the abstract an informative and balanced summary of what was done and what was found
**Introduction**		
Background/rationale	2<br>Page 2	Explain the scientific background and rationale for the investigation being reported
Objectives	3<br>Page 2	State specific objectives, including any prespecified hypotheses
**Methods**		
Study design	4<br>Page 3	Present key elements of study design early in the paper
Setting	5<br>Page 3	Describe the setting, locations, and relevant dates, including periods of recruitment, exposure, follow-up, and data collection

Participants	6<br>Pages 3 and 4	(*a*) Give the eligibility criteria, and the sources and methods of selection of participants. Describe methods of follow-up
		(*b*) For matched studies, give matching criteria and number of exposed and unexposed
Variables	7<br>Pages 4 and 5	Clearly define all outcomes, exposures, predictors, potential confounders, and effect modifiers. Give diagnostic criteria, if applicable
Data sources/ measurement	8*<br>Pages 4 and 5	For each variable of interest, give sources of data and details of methods of assessment (measurement). Describe comparability of assessment methods if there is more than one group
Bias	9<br>Page 6	Describe any efforts to address potential sources of bias
Study size	10<br>Page 4	Explain how the study size was arrived at
Quantitative variables	11<br>Page 6	Explain how quantitative variables were handled in the analyses. If applicable, describe which groupings were chosen and why
Statistical methods	12<br>Pages 4,5 and 6	(*a*) Describe all statistical methods, including those used to control for confounding
		(*b*) Describe any methods used to examine subgroups and interactions
		(*c*) Explain how missing data were addressed
		(*d*) If applicable, explain how loss to follow-up was addressed
		(*e*) Describe any sensitivity analyses
**Results**		
Participants	13*<br>Figure 1	(a) Report numbers of individuals at each stage of study—eg numbers potentially eligible, examined for eligibility, confirmed eligible, included in the study, completing follow-up, and analysed
		(b) Give reasons for non-participation at each stage
		(c) Consider use of a flow diagram
Descriptive data	14*<br>Table 1	(a) Give characteristics of study participants (eg demographic, clinical, social) and information on exposures and potential confounders

	Page 7	(b) Indicate number of participants with missing data for each variable of interest
		(c) Summarise follow-up time (eg, average and total amount)
Outcome data	15*<br>Pages 8,9 and 10	Report numbers of outcome events or summary measures over time
Main results	16<br>Pages 11,12 and 13	(*a*) Give unadjusted estimates and, if applicable, confounder-adjusted estimates and their precision (eg, 95% confidence interval). Make clear which confounders were adjusted for and why they were included
		(*b*) Report category boundaries when continuous variables were categorized
		(*c*) If relevant, consider translating estimates of relative risk into absolute risk for a meaningful time period
Other analyses	17<br>Supplementary tables 2 and 3	Report other analyses done—eg analyses of subgroups and interactions, and sensitivity analyses
**Discussion**		
Key results	18<br>Page 14	Summarise key results with reference to study objectives
Limitations	19<br>Page 16	Discuss limitations of the study, taking into account sources of potential bias or imprecision. Discuss both direction and magnitude of any potential bias
Interpretation	20<br>Pages 14 and 15	Give a cautious overall interpretation of results considering objectives, limitations, multiplicity of analyses, results from similar studies, and other relevant evidence
Generalisability	21<br>Page 16	Discuss the generalisability (external validity) of the study results
**Other information**		
Funding	22<br>Page 17	Give the source of funding and the role of the funders for the present study and, if applicable, for the original study on which the present article is based

*Give information separately for exposed and unexposed groups.

Note: An Explanation and Elaboration article discusses each checklist item and gives methodological background and published examples of transparent reporting. The STROBE checklist is best used in conjunction with this article (freely available on the Web sites of PLoS Medicine at http://www.plosmedicine.org/, Annals of Internal Medicine at http://www.annals.org/, and Epidemiology at http://www.epidem.com/). Information on the STROBE Initiative is available at http://www.strobe-statement.org.
